# The “sentinel stroke”: catheter ablation outcomes in patients with atrial fibrillation detected after stroke

**DOI:** 10.3389/fcvm.2026.1757625

**Published:** 2026-03-26

**Authors:** Yijie Liu, Jiawei Zhang, Yan Yin, Lili Wang, Qinchao Wu, Zhipeng Hu, Fenglin Qi, Yuhang Yang, Wenxi Dang, Sixian Weng, Yanguang Li, Xu Liu, Qiaoyuan Li, Ran Xiong, Tao Zhang, Zhuo Liang, Yunlong Wang

**Affiliations:** Department of Cardiology, Beijing Anzhen Hospital, Capital Medical University, Beijing, China

**Keywords:** atrial fibrillation, catheter ablation, ischemic stroke, post-stroke atrial fibrillation, propensity score matching

## Abstract

**Background:**

A subset of patients with atrial fibrillation (AF) first present with ischemic stroke. After neurological recovery, catheter ablation (CA) may be considered in arrhythmia clinic. However, outcomes of CA in patients with atrial fibrillation detected after stroke (AFDAS) remain insufficiently characterized compared with people with previously known AF (KAF).

**Methods:**

We conducted a retrospective analysis of consecutive patients who underwent catheter ablation between January 2023 and October 2024 in a special arrhythmia ward. Propensity score matching was performed to balance baseline characteristics. Outcome analyses were restricted to the matched cohort. The primary endpoint was freedom from atrial arrhythmia. The key secondary endpoint was a composite of all-cause death, stroke, or cardiovascular rehospitalization.

**Results:**

Among 3,417 eligible patients, 232 were included in the matched analysis (48 AFDAS, 184 KAF). AFDAS patients had numerically lower 2-year atrial arrhythmia recurrence rates [14.6% vs. 22.1%; HR 0.70, (95% CI 0.31–1.57); log-rank *P* = .39], although the difference was not statistically significant. The incidence of the key secondary endpoint was also similar between groups [9.2% vs. 14.1%; HR 0.73, (95% CI 0.28–1.90); log-rank *P* = .52].

**Conclusion:**

In this propensity score-matched cohort, CA was associated with comparable efficacy and safety outcomes in AFDAS and KAF patients. Larger prospective studies are needed to further clarify long-term outcomes in this population.

## Introduction

Atrial fibrillation (AF) is the most common sustained cardiac arrhythmia and a major contributor to ischemic stroke, heart failure, and cardiovascular mortality (1). AF increases the risk of ischemic stroke by approximately five-fold and is implicated in up to 25%–30% of acute ischemic stroke cases (2). With population aging and improved survival from cardiovascular disease, the global burden of AF continues to rise (3), underscoring the importance of effective strategies for stroke prevention and rhythm management (4).

A substantial proportion of patients with ischemic stroke have AF detected for the first time during or after the index cerebrovascular event. This clinical scenario, commonly referred to as **atrial fibrillation detected after stroke (AFDAS)**, reflects the growing use of prolonged electrocardiographic monitoring and heightened clinical vigilance following stroke (5, 6). In many cases, AF identified after stroke is paroxysmal, asymptomatic, or of low apparent burden, and would likely have remained undiagnosed in the absence of the cerebrovascular event (7, 8), raising questions regarding its temporal relationship to the index cerebrovascular event (5, 9). Whether such AF represents previously undiagnosed but causative arrhythmia, a marker of an underlying atrial cardiomyopathy, or a transient arrhythmia triggered by stroke-related autonomic or inflammatory mechanisms remains uncertain (9, 10). Accordingly, AFDAS is more appropriately viewed at present as a clinical mode of presentation rather than a mechanistically established arrhythmia subtype (5, 6, 9).

Accumulating evidence suggests that patients with AFDAS may differ from those with known atrial fibrillation (KAF) in terms of clinical characteristics and prognosis. Prior observational studies and meta-analyses have reported lower prevalences of structural heart disease and vascular comorbidities, as well as similar or lower risks of recurrent stroke and mortality, in patients with AFDAS compared with those with AF diagnosed before stroke (11–14). However, AFDAS is better understood at present as a clinical phenotype or mode of presentation rather than a mechanistically distinct arrhythmia (5, 6, 9). Considerable uncertainty remains regarding its underlying pathophysiology, including the relative contributions of previously undiagnosed AF, stroke-induced autonomic or inflammatory mechanisms, and atrial cardiomyopathy (9, 10). Within this framework, ischemic stroke can be conceptualized as a “sentinel event” that leads to the first clinical recognition of AF, thereby triggering cardiovascular evaluation and subsequent management. In our study, this “sentinel stroke” framework was used to define AFDAS as AF first identified in the context of an index ischemic stroke or TIA, without prior documented AF, emphasizing a mode of clinical presentation rather than a distinct biological entity.

Despite increasing interest in AFDAS, existing research has largely focused on stroke-related outcomes, such as recurrent cerebrovascular events, bleeding, and mortality (13–15), while comparatively little is known about arrhythmia-directed management in this population. In particular, whether rhythm-control strategies, including catheter ablation, confer comparable efficacy and safety in patients with AFDAS vs. those with known AF remains unclear. Addressing this knowledge gap, the present study aimed to compare baseline characteristics and post-ablation outcomes between patients with AFDAS and those with known AF undergoing catheter ablation, using propensity score–matched analyses in a real-world cohort.

## Methods

### Study population and definition

A retrospective analysis was performed using the medical records of consecutive patients who underwent CA in Beijing Anzhen Hospital, Arrhythmia Ward Three from January 2023 to October 2024. Adult patients with a principal discharge of AF (International Statistical Classification of Diseases and Related Health Problems 10th Revision, ICD-10, codes I48.x) who underwent CA were identified. Patients were prospectively entered into a database at the time of discharge, together with pertinent information and details about the ablation procedure. Had a patient had two or more admissions within the time window, the last admission during which CA was performed was included for the analysis. All patient data were de-identified and anonymized prior to analysis to ensure confidentiality. The Institutional Review Board of Beijing Anzhen Hospital granted an exemption for this study.

In accordance with prior observational studies (6) and to reflect real-world clinical practice, AFDAS was defined in this study as *atrial fibrillation first documented after the index ischemic stroke or transient ischemic attack in patients without a prior clinical history or electrocardiographic documentation of AF*. AF diagnosis was based on standard electrocardiographic criteria during routine clinical evaluation. Patients with previously documented AF before the index cerebrovascular event were classified as having known AF (KAF).

### Ablation procedure

All patients were on oral anticoagulants (OACs) (warfarin or novel OACs) for at least 3–4 weeks prior to the ablation procedure. Routine transesophageal echocardiography (TEE) or CT Angiography of Pulmonary Veins (PV-CTA) was performed prior to the ablation to exclude intra-atrial thrombosis. Most ablation procedures were performed with radiofrequency ablation and guided by a 3-dimensional electroanatomic mapping system (CARTO 3, Biosense Webster. EnSite Precision™ Cardiac Mapping System, Abbott), and followed a sequential approach. All patients underwent pulmonary vein isolation (PVI). Considering on left atrial size, AF duration, sex, age, etc., additional lesions could include isolation of the posterior wall (or LA roofline only), cavo-tricuspid isthmus (CTI) ablation, mitral isthmus (MI) ablation, superior vena cava isolation (SVCI) and other necessary lesions at the discretion of the operator. Additional ethanol infusion via the vein of Marshall (EIVOM) is predominantly adjunctive in persistent AF, with selective use in paroxysmal cases. If cryoballoon ablation was chosen, the procedure should strictly adhere to the standard protocol described in previous publications (16, 17). All ablation sites beyond PVI were recorded in the database.

Anticoagulation and antiarrhythmic drugs (AADs) were prescribed to all patients after CA for at least 3 months, and the continuation of anticoagulation and AADs were decided by physicians based on patients’ personal profiles. In most cases, AADs were discontinued at the end of the 3-month blanking period.

### Patient clinical characteristics and procedure-related data

We retrospectively collected data on baseline demographics, including age, sex, comorbidities, time of AF onset or duration of AF, length of hospital stay (LOHS), repeat ablation, cardiac laboratory data including left atrial diameter, left ventricular ejection fraction measured by transthoracic echocardiography (TTE), and CHA_2_DS-_2_VA score. CHA_2_DS_2_-VASc-60 stroke risk score (18) and C_2_HEST score (19) were also calculated for their capability in Asian/Chinese patients. The specific ablation strategy (additional linear ablation or low-voltage zone ablation), combining with left atrial appendage occlusion (LAAO) and left atrial volume were both retrospectively extracted from the procedural records. Complications, including perioperative ischemic stroke and cardiac tamponade, were captured from the medical records and relative physician orders.

The primary endpoint was freedom from documented atrial arrhythmia recurrence, defined as any episode of AF, atrial flutter, or atrial tachycardia lasting ≥30 s after a 3-month blanking period.

The key secondary endpoint was a composite of all-cause death, stroke, or cardiovascular rehospitalization (for heart failure or acute ischemic events). Additional secondary endpoints included recurrence of palpitation symptoms, continued use of AADs or anticoagulation.

All patients were instructed at discharge to undergo 12-lead ECG and 24 h Holter monitoring at 3 months post-ablation and annually thereafter. Additional ECG evaluation was performed if symptoms suggesting arrhythmia recurrence occurred. Annual telephone follow-up was conducted to ascertain clinical events. The last follow-up of this study was conducted in September 2025.

### Propensity score-matching and covariates

To reduce baseline differences between the AFDAS and KAF groups, a propensity score–matched analysis was performed. Propensity score matching was performed with a 1:4 ratio to reduce the imbalance of covariates between AFDAS and KAF groups. Age, gender, hypertension, diabetes, heart failure, coronary/peripheral artery disease, AF type (paroxysmal or persistent) was included in the model. Stroke or TIA were both excluded in the model due to collinearity with AFDAS. Matching was performed using the nearest neighbor matching protocol and a caliper width of 0.02, without using replacements. Covariate balance after matching was assessed using standardized mean differences, with values <0.1 considered indicative of adequate balance. Analyses were performed with STATA 18.0 (StataCorp, College Station, TX, USA).

### Statistical analysis

Continuous variables are presented as mean ± standard deviation (SD) and compared with Student's t tests. Categorical variables are presented as frequencies with percentages (%) and compared by the Chi-squared test or Fisher's exact test. All analyses are two-tailed, with statistical significance set at *p* < 0.05.

Cumulative incidences of primary and secondary endpoints were estimated by Kaplan–Meier survival curves and compared with log-rank tests. The effect of group (AFDAS vs. KAF) on the primary outcome is quantified using Cox regression, reported as hazard ratios (HRs). All-cause death was considered a competing event.

To reduce confounding, all time-to-event and clinical outcome analyses were restricted to the propensity score matched cohort.

## Results

### Demographics and comorbidities

From January 2023 to October 2024, a total of 3,472 hospital admissions for AF ablation were recorded. On the premise of retaining only the most recent ablation admission, 3417 unique patients were included in the final cohort, of whom 49 were classified as having AFDAS.

Baseline characteristics of the unmatched population are presented in Table 1. There were no significant differences between AFDAS and KAF patients in age, sex distribution, AF type, or major comorbidities. Patients with AFDAS had longer LOHS, higher stroke risk scores (CHA_2_DS_2_-VA or CHA_2_DS_2_-VASc−60) and were more likely to undergo LAAO, whereas additional linear ablation was more frequently performed in the KAF group. Severe complications (pericardial tamponade or effusion requiring pericardiocentesis) and ischemic stroke were rare (<1% each), with no in-hospital deaths during the index admission.

**Table 1:** Baseline characteristics of the unmatched patients.

Characteristic	Total *N* = 3,417	KAF group *N* = 3,368	AFDAS group *N* = 49	*P*-value
Demographics				
Sex (Female)	1,140 (33.4)	1,126 (33.4)	14 (28.6)	0.47
Age, mean (SD)	60.6 (10.0)	60.6 (10.0)	61.7 (9.3)	0.43
18–59 years	1,424 (41.7)	1,403 (41.7)	21 (42.9)	
60–64 years	641 (18.8)	638 (18.9)	3 (6.1)	
65–74 years	1,181 (34.6)	1,158 (34.4)	23 (46.9)	
≥75 years	171 (5.0)	169 (5.0)	2 (4.1)	
LOHS, median [IQR]	3 [2–3]	3 [2–3]	3 [2–4]	0.003
Persistent AF	1,238 (36.2)	1,220 (36.2)	18 (36.7)	0.94
Readmission	131 (3.8)	127 (3.8)	4 (8.2)	0.11
Comorbidities				
CHF	240 (7.0)	235 (7.0)	5 (10.2)	0.38
Hypertension	1,723 (7.0)	1,695 (50.3)	28 (57.1)	0.34
Diabetes mellitus	612 (17.9)	604 (17.9)	8 (16.3)	0.77
Stroke	257 (7.5)	208 (6.2)	49 (100.0)	<0.001
CAD	582 (17.0)	574 (17.0)	8 (16.3)	0.89
Hyperthyroidism	25 (0.7)	25 (0.7)	0 (0)	0.54
COPD	17 (0.5)	17 (0.5)	0 (0)	0.62
Ablation procedure				
Redo Procedure	254 (7.4)	252 (7.5)	2 (4.1)	0.37
LAAO	87 (2.5)	64 (1.9)	23 (46.9)	<0.001
Additional linear ablation	1,318 (38.6)	1,307 (38.8)	11 (22.4)	0.020
EIVOM	462 (13.5)	458 (13.6)	4 (8.2)	0.27
Major complication				
Cardiac tamponade	8 (0.2)	8 (0.2)	0 (0.0)	0.73
Perioperative ischemic stroke	3 (0.1)	3 (0.1)	0 (0.0)	0.83
Risk scores				
CHA_2_DS_2_-VA, median [IQR]	1 [0–2]	1 [0–2]	4 [3–4]	<0.001
0	898 (26.3)	898 (26.7)	0 (0.0)	<0.001
1	1,034 (30.3)	1,034 (30.7)	0 (0.0)	
≥2	1,485 (43.5)	1,436 (42.6)	49 (100.0)	
CHA_2_DS_2_-VASc-60, median [IQR]	2 [1–4]	2 [1–3]	5 [3–5]	<0.001
C_2_HEST, median [IQR]	0 [0–1]	0 [0–1]	0 [0–1]	0.43

After 1:4 propensity score matching, 48 patients with AFDAS were matched to 184 patients with KAF patients. Baseline covariates included in the matching model were well balanced between groups, with no significant differences in age, sex, comorbidities, or AF type (Table 2). Differences in stroke risk scores and LAAO utilization persisted, reflecting clinical characteristics associated with the index cerebrovascular event.

**Table 2:** Baseline characteristics of the matched patients^a^^,^^b^.

Characteristic	Total *N* = 232	KAF group *N* = 184	AFDAS group *N* = 48	*P*-value
Demographics				
Sex (Female)	79 (34.1)	65 (35.3)	14 (29.2)	0.42
Age, mean (SD)	61.6 (9.5)	61.6 (9.6)	61.5 (9.2)	0.93
≤44years	10 (4.3)	8 (4.3)	2 (4.2)	
45–54 years	55 (23.7)	44 (23.9)	11 (22.9)	
55–64 years	49 (21.1)	38 (20.7)	11 (22.9)	
65–74 years	108 (46.6)	86 (46.7)	22 (45.8)	
≥75years	10 (4.3)	8 (4.3)	2 (4.2)	
LOHS, median [IQR]	3 [2–3]	2 [2–3]	3 [2–4]	0.009
Persistent AF	90 (38.8)	72 (39.1)	18 (37.5)	0.84
TTE data				
LA, mean (SD)	40.8 (5.2)	40.7 (5.2)	41.3 (4.8)	0.54
LA enlarged	124 (53.4)	94 (56.3)	30 (63.8)	0.35
LVEF, mean (SD)	62.8 (6.4)	62.4 (6.5)	64.5 (6.0)	0.041
Comorbidities				
CHF	21 (9.1)	17 (9.2)	4 (8.3)	0.85
Hypertension	130 (56.0)	103 (56.0)	27 (56.2)	0.97
Diabetes mellitus	34 (14.7)	26 (14.1)	8 (16.7)	0.66
Stroke	60 (25.9)	12 (6.5)	48 (100.0)	<0.001
CAD	34 (14.7)	26 (14.1)	8 (16.7)	0.66
Ablation procedure				
Cryo	17 (7.3)	14 (7.7)	3 (6.2)	0.73
Redo Procedure	21 (9.1)	19 (10.3)	2 (4.2)	0.19
LAAO	26 (11.2)	4 (2.2)	22 (45.8)	<0.001
Additional linear ablation	82 (35.3)	70 (38.0)	12 (25.0)	0.092
CTI ablation	55 (23.7)	44 (24.0)	11 (22.9)	0.87
Roofline ablation	43 (18.5)	36 (19.7)	7 (14.6)	0.42
SVCI	34 (14.7)	31 (16.9)	3 (6.2)	0.063
MI ablation	31 (13.7)	26 (14.2)	5 (10.4)	0.49
LVA ablation	25 (10.8)	21 (11.5)	4 (8.3)	0.53
Redo ablation	4 (1.7)	3 (1.7)	1 (2.1)	0.85
EIVOM	27 (11.6)	23 (12.5)	5 (10.4)	0.69
Major complications				
Cardiac tamponade	2 (0.9)	2 (1.1)	0 (0.0)	0.47
Perioperative ischemic stroke	1 (0.4)	1 (0.5)	0 (0.0)	0.61
Readmission	11 (4.7)	7 (3.8)	4 (8.3)	0.19
CHA_2_DS_2_-VA, median [IQR]	2 [1–3]	1 [1–2]	4 [3–4]	<0.001
0	39 (16.8)	39 (21.2)	0 (0.0%)	
1	54 (23.3)	54 (29.3)	0 (0.0)	
≥2	139 (59.9)	91 (49.5)	48 (100.0)	
CHA_2_DS_2_VASc-60, median [IQR]	3 [2–4]	2 [1–4]	4.5 [3–5]	<0.001
Follow-up				
Death	2 (0.9)	2 (1.1)	0 (0.0)	0.47
Lost to follow-up	6 (2.6)	5 (2.7)	1 (2.1)	0.81
New-onset or recurrent stroke	3 (1.3)	2 (1.1)	1 (2.2)	
Continued anticoagulation	63 (27.2)	36 (20.3)	27 (60.0)	<0.001
Continued antiplatelet therapy	46 (19.8)	34 (19.2)	12 (26.7)	0.27
Continued AAD therapy	90 (38.8)	78 (43.8)	12 (26.7)	0.036
Palpitations	76 (33.2)	66 (38.3)	10 (21.3)	0.035

a Propensity score matching was performed using age, sex, hypertension, diabetes, heart failure, coronary/peripheral artery disease, and AF type (paroxysmal or persistent).

b Stroke or TIA were not included in the PSM model due to collinearity with AFDAS status.

### Primary outcome

Clinical outcomes were assessed in the propensity score-matched cohort.

After a median follow-up of 21.7 months (IQR 15.3–25.9), freedom from atrial arrhythmia was observed in 87.2% of AFDAS patients and 78.8% of KAF patients (Table 3). Two-year Kaplan–Meier event rates (after a 3-month blanking period) were 14.6% for AFDAS and 22.1% for KAF patients (Table 3).

**Table 3:** Primary and secondary clinical outcomes in propensity score-matched population^a^.

Characteristic	Events, no. (%)			*P*-value	Kaplan–Meier 2-year event rate, %		
	Total *N* = 232	KAF Group *N* = 184	AFDAS Group *N* = 48		KAF	AFDAS	Absolute reduction
Primary endpoint							
Atrial arrhythmia recurrence	44 (19.0)	38 (21.2)	6 (12.8)	0.19	22.1	14.6	7.5
Secondary endpoint							
Key secondary endpoint	31 (13.4)	26 (14.1)	5 (10.4)	0.50	15.3	10.5	4.8
Death	2 (0.9)	2 (1.1)	0 (0.0)	0.47			
New-onset or recurrent stroke	3 (1.3)	2 (1.1)	1 (2.2)	0.59			
Cardiovascular rehospitalization	29 (12.5)	24 (13.6)	5 (10.9)	0.62			
Palpitations	76 (33.2)	66 (38.3)	10 (21.3)	0.035			
Continued Anticoagulation	63 (27.2)	36 (20.3)	27 (60.0)	<0.001			
Continued antiplatelet therapy	46 (19.8)	34 (19.2)	12 (26.7)	0.27			
Continued AAD therapy	90 (38.8)	78 (43.8)	12 (26.7)	0.036			
Lost to follow-up	6 (2.6)	5 (2.7)	1 (2.1)	0.81			

a Due to the relatively low number of events, estimates should be interpreted with caution.

**Figure 1:**
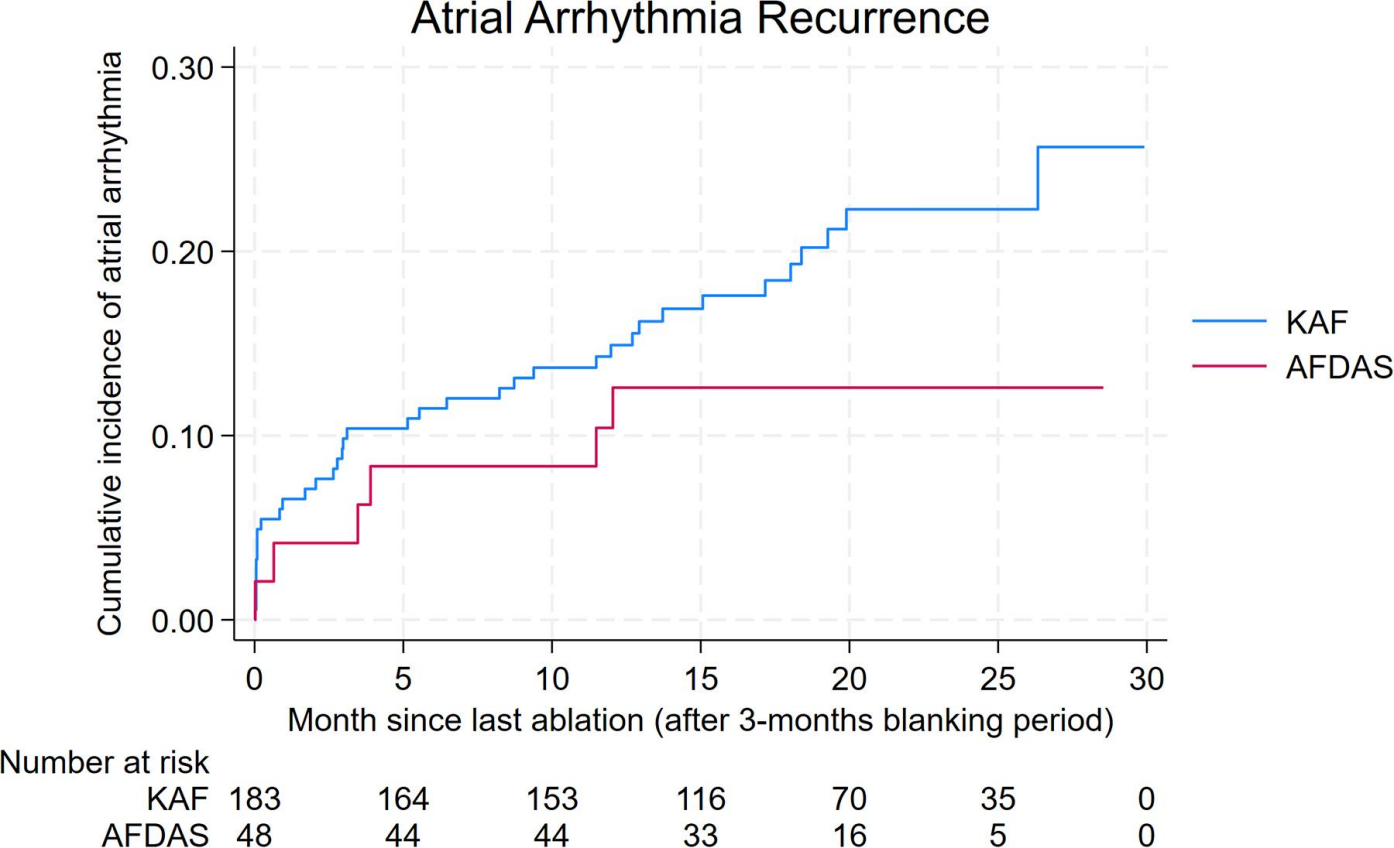
Kaplan–Meier curves for atrial arrhythmia recurrence.

No statistically significant difference in arrhythmia recurrence was observed between groups [HR 0.70; (95% CI 0.31–1.57); log-rank *P* = 0.39] (Figure 1). Results using the Fine-Gray proportional sub-distribution hazards model [sHR 0.70; (95% CI 0.31–1.57); *P* = 0.39] was consistent with the primary analysis. In multivariable Cox regression adjusting for baseline covariates, arrhythmia recurrence remained comparable between AFDAS and KAF patients [adjusted HR 1.20; (95% CI, 0.46–3.14); *P* = .70].

### Secondary outcomes

**Figure 2:**
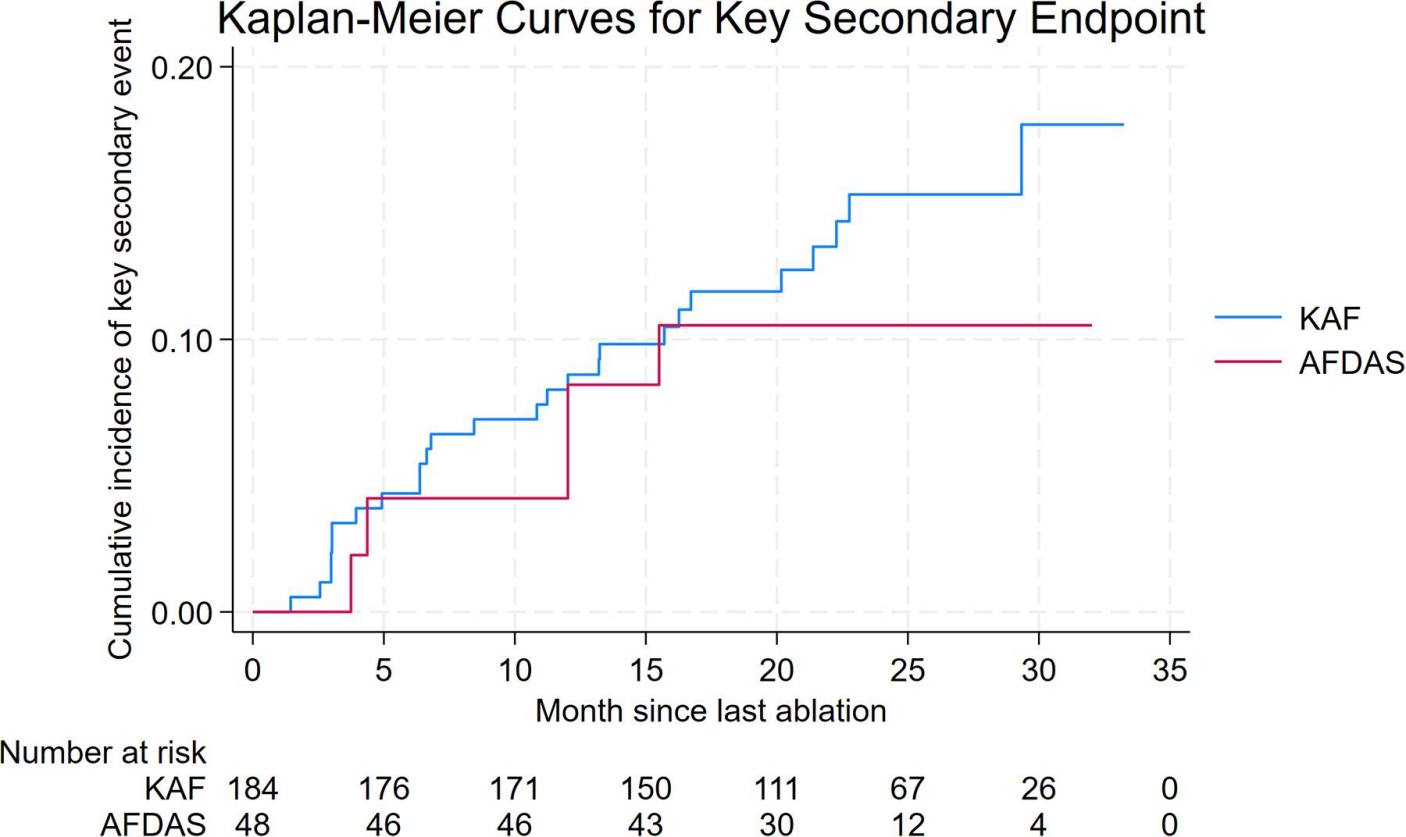
Kaplan–Meier estimates of the incidence of all-cause mortality, stroke and cardiovascular hospitalization.

In the propensity score-matched cohort, the key secondary endpoint event occurred in 26 KAF patients (14.1%) and 5 AFDAS patients (9.2%). No statistically significant difference was observed between groups [HR 0.73 (95% CI, 0.28–1.90); log-rank *P* = .52] (Figure 2). Two-year Kaplan–Meier event rates were 10.5% for AFDAS and 15.3% for KAF patients (Table 3).

Differences in post-procedural management were observed between groups. Patients with AFDAS were more likely to remain on anticoagulation therapy at follow-up (60.0% vs. 20.3%, *P* < 0.001). Conversely, patients with KAF more frequently reported palpitations (38.3% vs. 21.3%, *P* = 0.035) and were more frequently maintained on AADs, including β-blockers (43.8% vs. 26.7%, *P* = 0.036). The proportion of patients lost to follow-up was also low and comparable between groups (2.7% vs. 2.1%, *P* = 0.81).

### Subgroup analysis

**Figure 3:**
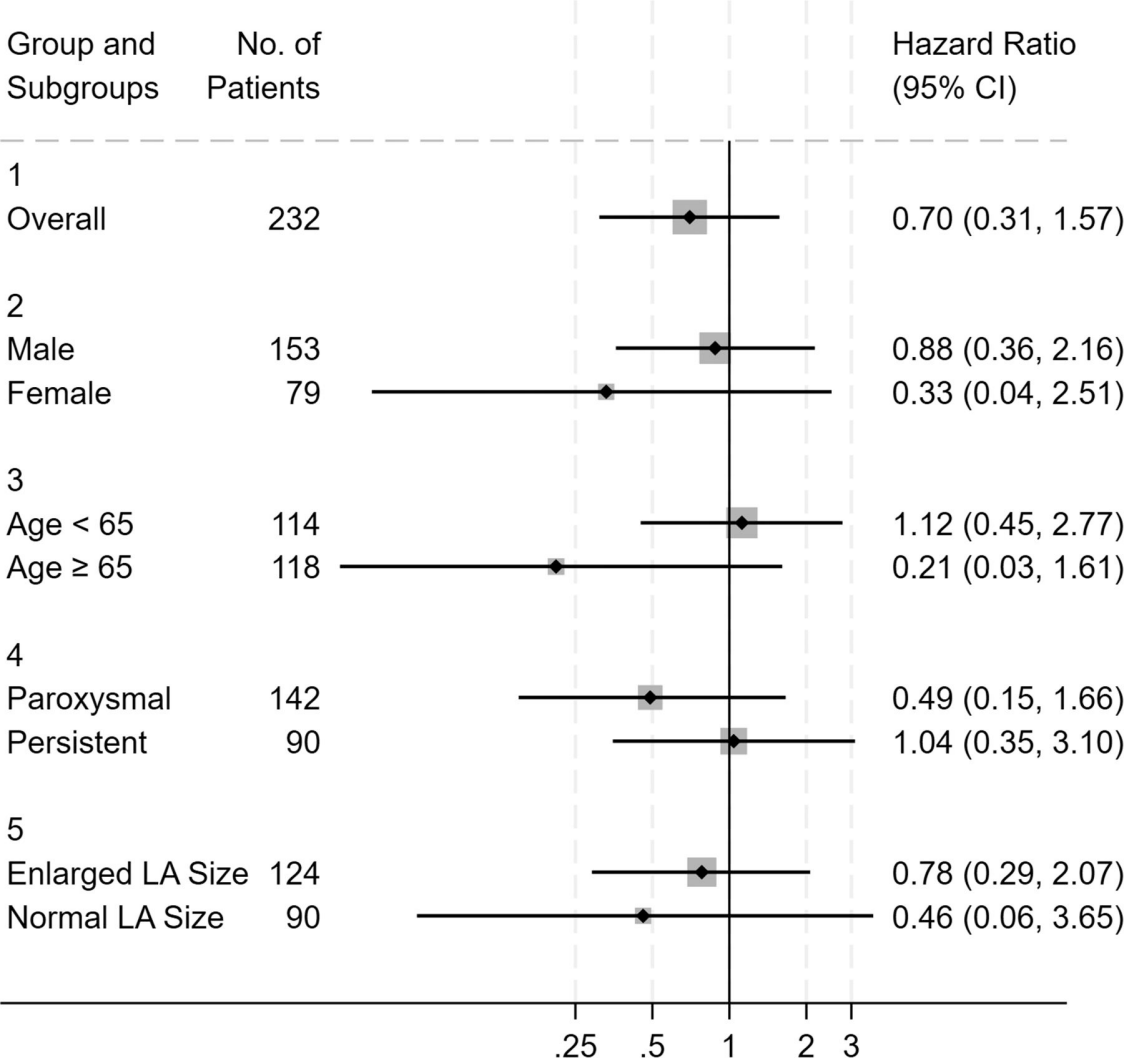
Sensitivity analysis for atrial arrhythmias recurrence during follow-up in matched population.

Exploratory subgroup analyses of atrial arrhythmias recurrence (by sex, age, left atrial diameter and types of AF) are shown in Figure 3. No statistically significant heterogeneity was observed across subgroups. Given the limited sample size and wide confidence intervals, these findings should be interpreted cautiously.

## Discussions

### Main findings

This propensity matched study firstly investigated the outcomes of CA in patients with atrial fibrillation diagnosed after stroke (AFDAS) compared to those with known atrial fibrillation (KAF). Arrhythmia recurrence rates were comparable between groups during follow-up, with no statistically significant difference observed in primary or competing-risk analyses. Similarly, the incidence of the composite secondary endpoint of death, stroke, or cardiovascular rehospitalization did not differ significantly between groups. Notable differences in post-ablation management were observed, including higher persistence of oral anticoagulation and lower reported palpitations among AFDAS patients. These findings suggest that, within a matched cohort undergoing ablation, AFDAS and KAF patients demonstrated broadly similar procedural and mid-term clinical outcomes.

Beyond procedural outcomes, our findings highlight differences in clinical profile and post-ablation management between AFDAS and KAF patients. In the matched cohort, AFDAS patients exhibited higher thromboembolic risk scores and were more frequently maintained on oral anticoagulation therapy, whereas KAF patients were more likely to receive additional antiarrhythmic medications and reported higher rates of symptomatic palpitations. These patterns suggest that AFDAS may represent a clinically distinct presentation of AF in the context of a recent cerebrovascular event, characterized by heightened attention to stroke prevention and structured follow-up.

### The role of CA in AFDAS patients

Previous studies on AFDAS have primarily focused on stroke-related outcomes, including recurrent ischemic events and mortality, with mixed findings compared to patients with known AF or sinus rhythm (SR) (13, 14). Some observational data reported a lower prevalence of heart disease in AFDAS compared to KAF (14), a positive association with functional independence in patients with AFDAS who underwent mechanical thrombectomy (15). However, evidence regarding rhythm-control strategies, particularly catheter ablation, in AFDAS patients remains limited (9).

Prolonged ECG monitoring (7) or insertable cardiac monitor (8) in survivors of stroke or TIA has improved AF detection rates and consequently increased referral of these patients to arrhythmia clinics. In this context, understanding the safety and effectiveness of catheter ablation in AFDAS has practical clinical relevance. In the present propensity score-matched cohort, arrhythmia recurrence and major clinical outcomes were comparable between AFDAS and KAF patients.

Importantly, given the observational design and limited number of events, our results should not be interpreted as evidence of superiority but rather as supportive of the feasibility and mid-term safety of ablation in carefully selected AFDAS patients. Further prospective studies are required to clarify whether rhythm-control strategies confer differential long-term benefits in this population.

### Sentinel stroke and clinical presentation of AFDAS

The concept of “sentinel stroke” provides a useful framework for understanding AFDAS within the spectrum of atrial fibrillation presentations. In this study, we applied this concept as *ischemic stroke or TIA serving as the first clinical manifestation leading to AF detection in patients without prior documented AF, regardless of AF type (paroxysmal or persistent)*. This distinction separates AFDAS from clinically recognized AF preceding stroke (KAF), and formed the basis of our patient classification.

From a pathophysiological perspective, the temporal relationship between stroke and newly detected AF remains debated, with proposed mechanisms including pre-existing but previously undiagnosed AF, stroke-triggered autonomic dysregulation, and broader stroke-heart interactions (9, 10). Regardless of the underlying mechanism, the occurrence of stroke as the first clinical manifestation of AF represents a clinically significant entry point into cardiovascular care. In our cohort, this “sentinel” presentation was associated with higher thromboembolic risk scores and greater persistence of anticoagulation therapy during follow-up, reflecting a management pathway strongly oriented toward secondary stroke prevention.

While the sentinel stroke concept informed our classification strategy and interpretation of management patterns, our data do not allow determination of causality between stroke and AF onset. Rather, this framework serves to contextualize AFDAS as a distinct clinical presentation that may require tailored longitudinal management, which warrants further investigation in prospective studies.

### Mechanistic considerations of AFDAS

Several mechanistic hypotheses may be considered when interpreting AFDAS within the broader context of atrial disease. Atrial cardiomyopathy (AtCM) and AF are closely interrelated, and stroke occurring in association with newly detected AF has been proposed as a potential manifestation of underlying atrial structural or functional abnormalities (20, 21). AF itself may promote atrial remodeling through a self-perpetuating process (“AF begets AF”) (22, 23).

In addition, transient atrial mechanical dysfunction (“atrial stunning”) following AF termination has been implicated in thromboembolic risk (24, 25), with or without using anticoagulation after cardioversion (26, 27). Whether such mechanisms contribute to the temporal relationship between stroke and AF detection in AFDAS remains uncertain. The present study did not directly assess structural remodeling, atrial mechanical function, or biomarkers of atrial cardiomyopathy so that no mechanistic conclusions can be drawn. These considerations should be regarded as theoretical and hypothesis-generating. Further mechanistic and longitudinal studies are required to clarify the pathophysiological underpinnings of AFDAS.

### Post-ablation medical management for AFDAS patients

Differences in post-ablation medical management were observed between groups. AFDAS patients were more frequently maintained on oral anticoagulation and more often underwent left atrial appendage occlusion, likely reflecting their higher baseline thromboembolic risk profiles and prior cerebrovascular events (28). In contrast, KAF patients more commonly reported palpitations and were more frequently treated with antiarrhythmic drugs during follow-up.

These differences in management strategies may have influenced clinical outcomes and should be considered when interpreting between-group comparisons. Although recent studies have explored anticoagulation discontinuation after successful AF ablation (29), the optimal long-term antithrombotic strategy in AFDAS remains uncertain. Given the observational design and limited number of events in the present study, no definitive recommendations regarding anticoagulation continuation or LAAO strategies can be made. Prospective studies are required to determine the most appropriate post-ablation management approach in this population.

### Limitations

This study has several important limitations. First, as a retrospective, single-center observational study, residual confounding and selection bias cannot be excluded despite propensity score matching. The relatively small number of AFDAS patients and low event rates limited statistical power; therefore, the absence of statistically significant differences should be interpreted as inconclusive rather than evidence of no effect.

Second, the study population consisted of hospitalized patients undergoing catheter ablation. AFDAS patients managed exclusively in outpatient settings or those with more severe neurological impairment who were not referred for ablation were not captured, which may limit generalizability.

Third, detailed data regarding AF burden, precise AF onset timing, and stroke-AF temporal sequencing were not systematically available. Consequently, more granular analyses of AF characteristics and their relationship to outcomes could not be performed.

Fourth, definitions of AFDAS have varied across prior studies. We adopted a clinically pragmatic definition based on first documentation of AF after index ischemic stroke or TIA. Although this approach reflects real-world practice, differences in definitions may limit cross-study comparability.

Fifth, although the safety and efficacy of pulse-field ablation (PFA) are well-established (30–32), PFA was not available at our center during the study period. Accordingly, the present findings pertain to conventional ablation techniques and should be interpreted within that technological context.

Finally, mechanistic variables such as imaging markers of atrial remodeling or biomarkers of atrial cardiomyopathy were not assessed, precluding mechanistic inference.

## Conclusion

In this propensity score-matched cohort, catheter ablation in patients with atrial fibrillation diagnosed after stroke (AFDAS) was associated with outcomes comparable to those observed in patients with previously known atrial fibrillation (KAF). No significant differences were detected in arrhythmia recurrence or major adverse clinical events. These findings support the concept that AFDAS represents a distinct clinical phenotype, characterized by specific clinical features and post-ablation management patterns, with a clinical course broadly similar to that of patients with KAF following ablation.

However, given the limited statistical power and observational design, the present results should be considered hypothesis-generating. Prospective, multicenter investigations are warranted to further clarify the long-term prognosis and post-ablation management strategies in this population.

## Data Availability

The datasets presented in this article are not readily available because of the institutional data policies designed to ensure participant privacy. Requests to access the datasets should be directed to yj-sage@qq.com.

## References

[1] Ko D Chung MK Evans PT Benjamin EJ Helm RH. Atrial fibrillation: a review. JAMA. (2025) 333(4):329–42. 10.1001/jama.2024.22451 39680399 PMC11774664

[2] Lip GYH Proietti M Potpara T Mansour M Savelieva I Tse HF Atrial fibrillation and stroke prevention: 25 years of research at EP europace journal. Europace. (2023) 25(9):1–38. 10.1093/europace/euad226 37622590 PMC10451006

[3] Cheng S He J Han Y Han S Li P Liao H Global burden of atrial fibrillation/atrial flutter and its attributable risk factors from 1990 to 2021. Europace. (2024) 26(7):1–20. 10.1093/europace/euae195 PMC11287210 38984719

[4] Schnabel RB Yin X Gona P Larson MG Beiser AS McManus DD 50 Year trends in atrial fibrillation prevalence, incidence, risk factors, and mortality in the framingham heart study: a cohort study. Lancet. (2015) 386(9989):154–62. 10.1016/S0140-6736(14)61774-8 25960110 PMC4553037

[5] Sposato LA Field TS Schnabel RB Wachter R Andrade JG Hill MD. Towards a new classification of atrial fibrillation detected after a stroke or a transient ischaemic attack. Lancet Neurol. (2024) 23(1):110–22. 10.1016/S1474-4422(23)00326-5 37839436

[6] Sposato LA Chaturvedi S Hsieh CY Morillo CA Kamel H. Atrial fibrillation detected after stroke and transient ischemic attack: a novel clinical concept challenging current views. Stroke. (2022) 53(3):e94–e103. 10.1161/STROKEAHA.121.034777 34986652

[7] Grond M Jauss M Hamann G Stark E Veltkamp R Nabavi D Improved detection of silent atrial fibrillation using 72-hour holter ECG in patients with ischemic stroke: a prospective multicenter cohort study. Stroke. (2013) 44(12):3357–64. 10.1161/STROKEAHA.113.001884 24130137

[8] Healey JS Connolly SJ Gold MR Israel CW Van Gelder IC Capucci A Subclinical atrial fibrillation and the risk of stroke. N Engl J Med. (2012) 366(2):120–9. 10.1056/NEJMoa1105575 22236222

[9] Sposato LA Lip GYH Haeusler KG. Atrial fibrillation first detected after stroke: is timing and detection intensity relevant for stroke risk? Eur Heart J. (2024) 45(5):396–8. 10.1093/eurheartj/ehad744 38014646

[10] Seiffge DJ Cancelloni V Räber L Paciaroni M Metzner A Kirchhof P Secondary stroke prevention in people with atrial fibrillation: treatments and trials. Lancet Neurol. (2024) 23(4):404–17. 10.1016/S1474-4422(24)00037-1 38508836

[11] Fridman S Jimenez-Ruiz A Vargas-Gonzalez JC Sposato LA. Differences between atrial fibrillation detected before and after stroke and TIA: a systematic review and meta-analysis. Cerebrovasc Dis. (2022) 51(2):152–7. 10.1159/000520101 34844239

[12] Romoli M Urbinati G Tudisco V Toscano A Eusebi P Giammello F Risk of recurrent stroke, mortality, and intracerebral hemorrhage in patients with atrial fibrillation detected before or after a stroke. Neurology. (2025) 104(6):e213426. 10.1212/WNL.0000000000213426 39999395

[13] Yang XM Rao ZZ Gu HQ Zhao XQ Wang CJ Liu LP Atrial fibrillation known before or detected after stroke share similar risk of ischemic stroke recurrence and death. Stroke. (2019) 50(5):1124–9. 10.1161/STROKEAHA.118.024176 31009353

[14] Sposato LA Cerasuolo JO Cipriano LE Fang J Fridman S Paquet M Atrial fibrillation detected after stroke is related to a low risk of ischemic stroke recurrence. Neurology. (2018) 90(11):e924–e31. 10.1212/WNL.0000000000005126 29444969

[15] Ryu JC Lee SH Jung JM Kwon B Song Y Lee DH Association between the timing of atrial fibrillation detection and functional outcome following mechanical thrombectomy. J Am Heart Assoc. (2024) 13(17):e034861. 10.1161/JAHA.124.034861 39190593 PMC11646505

[16] Kuniss M Pavlovic N Velagic V Hermida JS Healey S Arena G Cryoballoon ablation vs. Antiarrhythmic drugs: first-line therapy for patients with paroxysmal atrial fibrillation. Europace. (2021) 23(7):1033–41. 10.1093/europace/euab029 33728429 PMC8286851

[17] Hoffmann E Straube F Wegscheider K Kuniss M Andresen D Wu LQ Outcomes of cryoballoon or radiofrequency ablation in symptomatic paroxysmal or persistent atrial fibrillation. Europace. (2019) 21(9):1313–24. 10.1093/europace/euz155 31199860 PMC6735953

[18] Ma C Wu S Liu S Han Y. Chinese Guidelines for the diagnosis and management of atrial fibrillation. Pacing Clin Electrophysiol. (2024) 47(6):714–70. 10.1111/pace.14920 38687179

[19] Li YG Liu YJ Wang LL Li QY Zhang T Liu X Dynamic increase of the C(2)HEST score in relation to the development of incident atrial fibrillation: a longitudinal cohort study. J Am Heart Assoc. (2025) 14(19):e039231. 10.1161/JAHA.124.039231 40970537 PMC12684495

[20] Kittipibul V Laufer-Perl M Balakumaran K Costanzo MR Marwick TH Alenezi F Atrial mechanics, atrial cardiomyopathy and impact of atrial interventions. J Card Fail. (2024) 30(10):1355–66. 10.1016/j.cardfail.2024.06.017 39389746

[21] Guichard JB Nattel S. Atrial cardiomyopathy: a useful notion in cardiac disease management or a passing fad? J Am Coll Cardiol. (2017) 70(6):756–65. 10.1016/j.jacc.2017.06.033 28774383

[22] Andrade J Khairy P Dobrev D Nattel S. The clinical profile and pathophysiology of atrial fibrillation: relationships among clinical features, epidemiology, and mechanisms. Circ Res. (2014) 114(9):1453–68. 10.1161/CIRCRESAHA.114.303211 24763464

[23] Nattel S Harada M. Atrial remodeling and atrial fibrillation: recent advances and translational perspectives. J Am Coll Cardiol. (2014) 63(22):2335–45. 10.1016/j.jacc.2014.02.555 24613319

[24] Ding WY Gupta D Lip GYH. Atrial fibrillation and the prothrombotic state: revisiting virchow’s triad in 2020. Heart. (2020) 106(19):1463–8. 10.1136/heartjnl-2020-316977 32675218

[25] Khan IA. Transient atrial mechanical dysfunction (stunning) after cardioversion of atrial fibrillation and flutter. Am Heart J. (2002) 144(1):11–22. 10.1067/mhj.2002.123113 12094183

[26] Airaksinen KE Grönberg T Nuotio I Nikkinen M Ylitalo A Biancari F Thromboembolic complications after cardioversion of acute atrial fibrillation: the FinCV (Finnish CardioVersion) study. J Am Coll Cardiol. (2013) 62(13):1187–92. 10.1016/j.jacc.2013.04.089 23850908

[27] Itäinen-Strömberg S Lehto M Halminen O Putaala J Haukka J Lehtonen O Thromboembolic and bleeding complications after elective cardioversion of atrial fibrillation: a nationwide cohort study. Europace. (2024) 26(6):1–10. 10.1093/europace/euae131 PMC11146156 38829189

[28] Osmancik P Herman D Neuzil P Hala P Taborsky M Kala P 4-Year Outcomes after left atrial appendage closure versus nonwarfarin oral anticoagulation for atrial fibrillation. J Am Coll Cardiol. (2022) 79(1):1–14. 10.1016/j.jacc.2021.10.023 34748929

[29] Kim D Shim J Choi EK Oh IY Kim J Lee YS Long-Term anticoagulation discontinuation after catheter ablation for atrial fibrillation: the ALONE-AF randomized clinical trial. Jama. (2025) 334(14):1246–54. 10.1001/jama.2025.14679 40886309 PMC12400166

[30] Reddy VY Gerstenfeld EP Natale A Whang W Cuoco FA Patel C Pulsed field or conventional thermal ablation for paroxysmal atrial fibrillation. N Engl J Med. (2023) 389(18):1660–71. 10.1056/NEJMoa2307291 37634148

[31] Ekanem E Neuzil P Reichlin T Kautzner J van der Voort P Jais P Safety of pulsed field ablation in more than 17,000 patients with atrial fibrillation in the MANIFEST-17K study. Nat Med. (2024) 30(7):2020–9. 10.1038/s41591-024-03114-3 38977913 PMC11271404

[32] Turagam MK Neuzil P Schmidt B Reichlin T Neven K Metzner A Safety and effectiveness of pulsed field ablation to treat atrial fibrillation: one-year outcomes from the MANIFEST-PF registry. Circulation. (2023) 148(1):35–46. 10.1161/CIRCULATIONAHA.123.064959 37199171

